# Odd Chain Fatty Acids Are Not Robust Biomarkers for Dietary Intake of Fiber

**DOI:** 10.1002/mnfr.202100316

**Published:** 2021-10-22

**Authors:** Yiwei Wu, Joram M. Posma, Elaine Holmes, Gary Frost, Edward S. Chambers, Isabel Garcia‐Perez

**Affiliations:** ^1^ Department of Metabolism Digestion and Reproduction Faculty of Medicine Imperial College London London UK; ^2^ Division of Systems Medicine Department of Metabolism Digestion and Reproduction Faculty of Medicine Imperial College London London UK; ^3^ Health Data Research UK‐London London UK

**Keywords:** biomarker validation, dietary biomarker, dietary fiber, odd chain fatty acids, short chain fatty acids

## Abstract

**Scope:**

Prior investigation has suggested a positive association between increased colonic propionate production and circulating odd‐chain fatty acids (OCFAs; pentadecanoic acid [C15:0], heptadecanoic acid [C17:0]). As the major source of propionate in humans is the microbial fermentation of dietary fiber, OCFAs have been proposed as candidate biomarkers of dietary fiber. The objective of this study is to critically assess the plausibility, robustness, reliability, dose‐response, time‐response aspects of OCFAs as potential biomarkers of fermentable fibers in two independent studies using a validated analytical method.

**Methods and Results:**

OCFAs are first assessed in a fiber supplementation study, where 21 participants received 10 g dietary fiber supplementation for 7 days. OCFAs are then assessed in a highly controlled inpatient setting, which 19 participants consumed a high fiber (45.1 g per day) and a low fiber diet (13.6 g per day) for 4 days. Collectively in both studies, dietary intakes of fiber as fiber supplementations or having consumed a high fiber diet do not increase circulating levels of OCFAs. The dose and temporal relations are not observed.

**Conclusion:**

Current study has generated new insight on the utility of OCFAs as fiber biomarkers and highlighted the importance of critical assessment of candidate biomarkers before application.

## Graphical abstract

Odd chain fatty acids (OCFAs) have been suggested as candidate biomarkers of dietary fiber. However, assessment of their validity has not been done. This study evaluated the key aspects of OCFAs as fiber biomarkers in two independent studies. This work showed that high fiber intakes did not increase circulating levels of OCFAs, highlighting the importance of biomarker validation prior application.

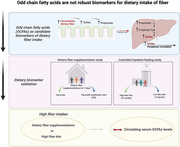

## 1 Introduction

Dietary fiber is the collective term for carbohydrate polymers with 10 or more monomeric units that escape digestion and absorption in the small intestine.^[^ ^1^ ^]^ The intake of dietary fiber, especially food high in fermentable fibers such as pectin and inulin‐type fructans has been associated with increased colonic short chain fatty acids (SCFAs) production by the gut bacteria.^[^ ^2^, ^3^ ^]^ The beneficial roles of dietary fiber have been implicated in many interventional and observational studies.^[^ ^4^ ^]^ However, studies investigating the health impacts of dietary fiber have relied on self‐reported methods such as diet history, 24‐h dietary recall and food frequency questionnaire. The traditional dietary assessment methods have been widely used and inherently associated with random errors and bias.^[^ ^5^, ^6^ ^]^ It has been estimated that approximately 30–88% of the adults’ misreport dietary information,^[^ ^7^, ^8^ ^]^ which confronts the understanding of true health impact of dietary fiber, or any dietary interventions aimed at influencing food behavior change. Thus, attempts to assess health benefits of dietary fiber require accurate intake assessment.

The application of metabolic profiling to identify new biomarkers of specific nutrient, food group, or dietary pattern has risen rapidly over the past decade.^[^ ^9^, ^10^, ^11^ ^]^ Dietary biomarkers are metabolites directly or indirectly derived from food or food constituent that are measurable in the biofluids over a fixed period of time.^[^ ^12^ ^]^ Although a great variety of putative dietary biomarkers have been identified, the numbers that have been comprehensively validated are still limited.^[^ ^13^, ^14^ ^]^ For instance, total urinary nitrogen for protein intake, vitamin C for fruits and vegetables, alkylresorcinols as biomarkers of whole grain wheat and rye intake.^[^ ^15^, ^16^, ^17^, ^18^ ^]^ In fact, only 23% of the biomarkers proposed in initial study have been successfully validated in the follow‐up investigation.^[^ ^19^ ^]^ A biomarker validation scheme, this includes the assessment of biomarker plausibility, robustness, reliability, dose‐response, time‐response, stability, analytical performance, and reproducibility has been recently proposed by Dragsted et al.^[^ ^20^ ^]^ Following the proposed scheme could avoid the use of putative biomarkers that without further validation may lead to misinterpretation and inaccurate findings.

With the continuous interest in dietary fiber, putative biomarkers have been proposed in observational and dietary intervention studies.^[^ ^21^, ^22^, ^23^ ^]^ Weitkunat et al.^[^ ^23^ ^]^ first proposed OCFAs, specifically, pentadecanoic acid (C15:0) and heptadecanoic acid (C17:0) as candidate biomarkers of dietary fiber. In this study, participants received cellulose, inulin, and sodium propionate for 7 days. Cellulose, a poorly fermentable dietary fiber did not lead to significant changes in OCFAs levels. However, inulin and propionate supplementations significantly increased plasma OCFAs with pronounced effect observed between propionate and C17:0. Of note, OCFAs have been considered as well functioning biomarkers of dairy fat intake in adipose tissue and blood lipid fractions.^[^ ^24^, ^25^, ^26^, ^27^ ^]^ Therefore, differentiating dietary sources of OCFAs in a complex meal could be challenging. Here, we apply the validation criteria proposed by Dragsted et al.^[^ ^20^ ^]^ to evaluate the applicability of OCFAs as biomarkers of dietary fiber intake in two laboratory based clinical studies.

## 2 Experimental Section

### 2.1 The Dietary Fiber Supplementation Intervention Trial (Study 1)

**Figure 1:**
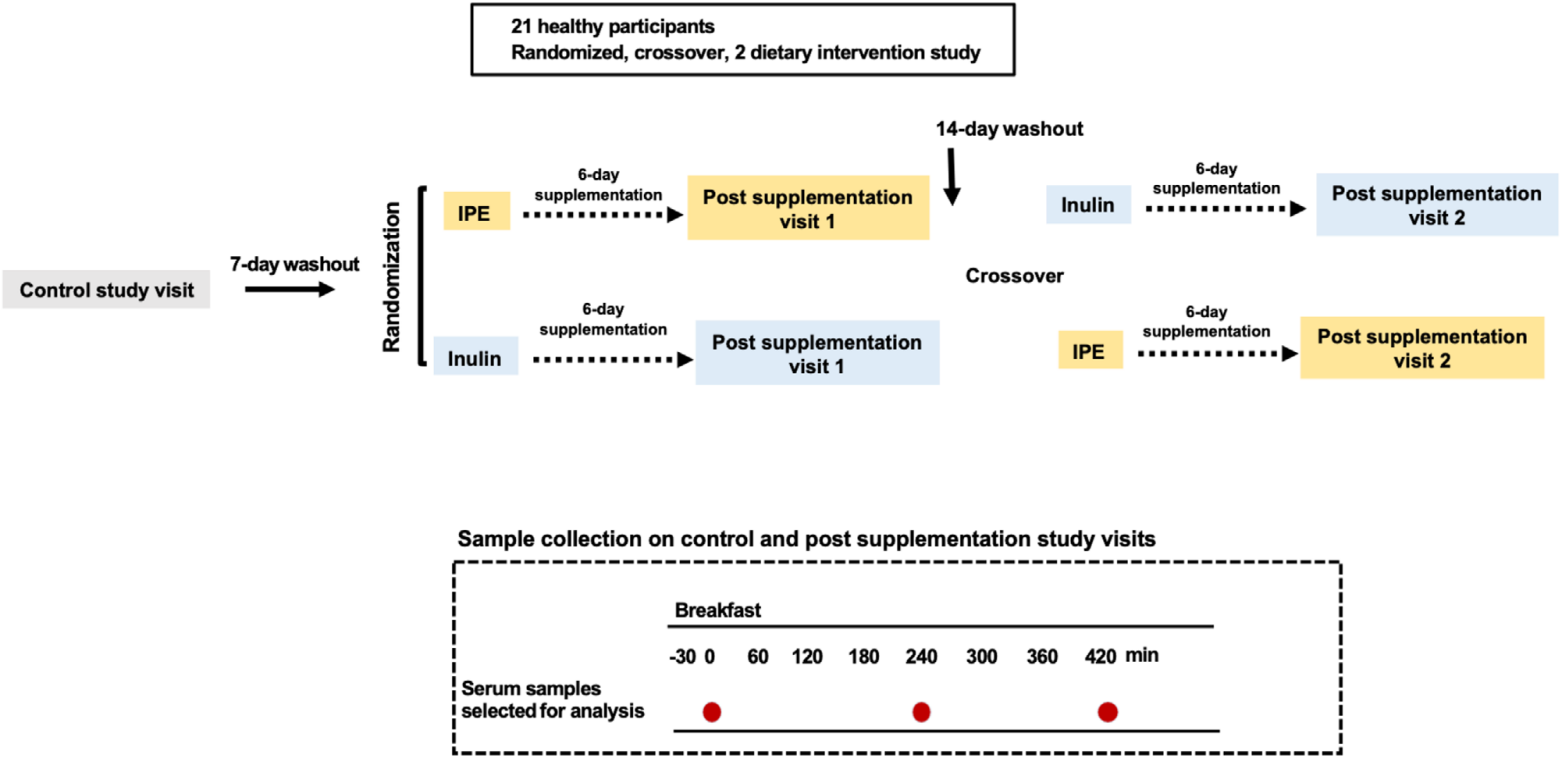
Study design for the dietary fiber supplementation intervention trial (Study 1), (*n* = 21). Participants first underwent a control study visit and received a standard breakfast without supplementation. Following 7‐day washout, in a randomized crossover manner, participants received either 10 g per day inulin or IPE supplementation for 6 days leading to a post‐supplementation study visit on day‐7. On each post‐supplementation study visit, a standardized breakfast contain 10 g inulin or IPE were assigned to the participants based on randomization. The postprandial dietary response was monitored up to 420 min. The blood samples obtained at 0, 240, and 420 min were selected for analysis.

This was a randomized, double‐blind, crossover, 7‐day supplementation study. All participants provided informed, written consent prior to the study. This study was approved by the London‐Brent REC (REC Reference Number 14/LO/0645) and registered on the ISRCTN registry (ISRCTN71814178, http://www.isrctn.com/ISRCTN71814178). The study conformed to the Declaration of Helsinki of 1975 and later revisions. Details regarding participant recruitment, eligibility, and study design have been published elsewhere.^[^ ^28^ ^]^ Briefly, 21 healthy participants were first asked to complete a control study visit having consumed a standard breakfast without fiber supplementations. In a randomized crossover design, participants were randomly assigned to receive (1) inulin (10 g per day), a polymer of fructose belongs to the category of dietary fiber with high fermentability, (2) inulin propionate ester (IPE, 10 g per day), a novel dietary molecule that was designed for targeted delivery of propionate into the colon.^[^ ^29^ ^]^ The dietary fiber supplementations were consumed as part of participants' habitual diet for 6 days. On day 7, participants completed a post‐supplementation study visit. On the post‐supplementation study visit, participants received a standard breakfast that contained 10 g inulin or IPE as acute feeding investigation for 420 min (**Figure** **1**). Blood samples were collected at time 0, 240 and 420 min post ingestion in control study visit, post supplementation study visits 1 and 2. 10 mL blood was collected at each timepoint, serum tubes were allowed to clot before centrifugation (BD Serum SST Vacutainer). Resulting serum was aliquoted into Eppendorf's and frozen at –80 °C until analysis.

### 2.2 The Highly Controlled in‐Patient Feeding Study (Study 2)

**Figure 2:**
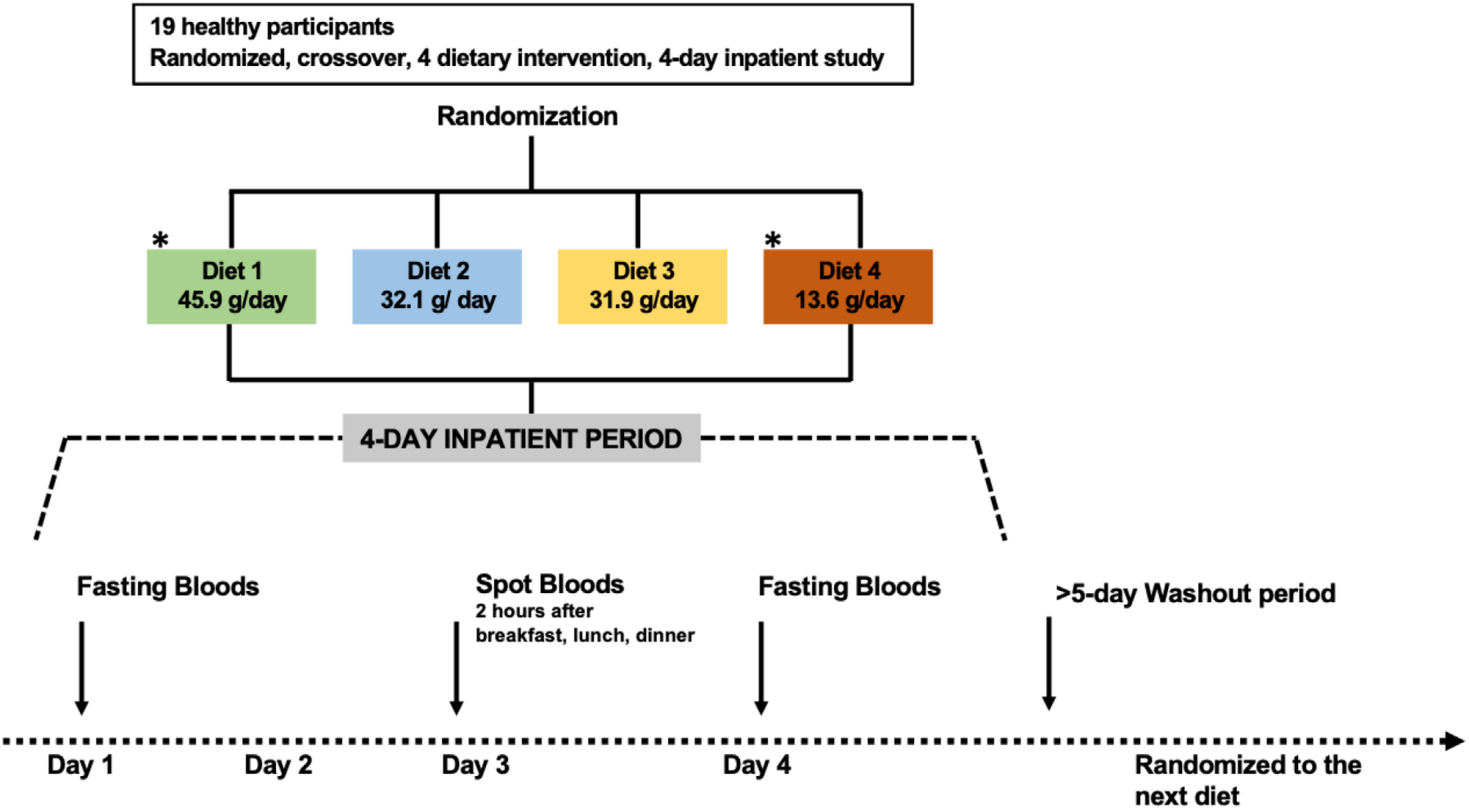
Study design for the highly controlled in‐patient feeding study (Study 2), (*n* = 19). In a randomized, crossover manner, four separate study visits were carried out in this study with a washout period more than 5 days between each visit. The fasting serum samples were collected on the first and fourth day of the study period, and the spot serum samples were collected 2 h after breakfast, lunch, and dinner on day‐3 of the investigation. *Samples obtained from Diet 1 and Diet 4 as more extreme representation of high or low fiber diet were analyzed in present work.

This was a highly controlled, inpatient, randomized, crossover clinical trial. All participants were provided with informed, written consent prior to the study. This study was approved by the London‐Brent REC (REC Reference Number 13/LO/0078) and registered on the ISRCTN registry (ISRCTN43087333, http://www.isrctn.com/ISRCTN43087333). The study conformed to the Declaration of Helsinki of 1975 and later revisions. Details regarding participant recruitment, eligibility, and study design have been published elsewhere.^[^ ^30^ ^]^ Briefly, 19 participants were randomly assigned to four types of diet with differing levels of adherence to World Health Organization dietary guidelines: two extreme diets and two intermediate diets. Each diet consists of three main meals and two snacks per day and was followed by participants under a controlled environment for a 4‐day inpatient period (**Figure** **2**). There were no differences in the fiber content of the intermediate diets (Diet 2 = 32.1 g per day, Diet 3 = 31.9 g per day) but the two extreme diets contained different amounts of dietary fiber (Diet 1 = 45.1 g per day and Diet 4 = 13.6 g per day), therefore selected for this study. Further information on the dietary information and nutritional profile of four types of diets can be found in Table S1, Supporting Information. The fasting blood samples were collected at Day 1 and the end Day 4 of each diet. Spot blood samples were collected on the Day 3 of each diet, 2 h after breakfast, 2 h after lunch, and 2 h after dinner. Blood samples were collected by a trained nurse using two vacutainers gel tubes of 5 mL. These tubes were inverted five times after collection, placed horizontally on ice and spin at 3100 x *g* for 15 min at 4 °C. The resulting serum was decanted from the tube and stored immediately at −80 °C until analysis.

### 2.3 Sample Preparation

The standard solution of C15:0 and C17:0 from 0.1 to 300 ppm was used for the preparation of calibration curves in triplicates for C15:0 and C17:0 quantification. Sample from both studies were prepared following the extraction and derivatization protocol previously developed by Zhao et al.^[^ ^31^ ^]^ Each aliquoted 100 µL serum sample was extracted with 300 µL cold methanol for protein precipitation and placed at −40 °C for 30 min. Each sample was centrifuged at 14,000 × *g*, 4 °C for 10 min, the supernatant was immediately transferred to an autosampler glass vial for evaporation. The supernatant was evaporated using Speedvac for methanol removal at 30 °C, 14,000 × *g* for 1.5 h and stored at –40 °C for subsequent derivatization steps. The sealed vial containing serum sample or calibration standard after evaporation was placed on ice and defrosted. Each sample was re‐dissolved in 200 µL sodium hydroxide (1M) at 4 °C and mixed with 167 µL methanol and 34 µL pyridine. 20 µL ethyl chloroformate (ECF) was added to each sample mixture followed by vortex for 30 s. Another 20 µL of ECF was added to each sample mixture followed by vortex for 30 s. Subsequently, 400 µL of mixture, containing 120 µL of internal standard (methyl stearate) and 280 µL of chloroform was added to the sample mixture, followed by vortex for 10 s. Thereafter, 400 µL sodium bicarbonate solution (50 mM) was added to each sample mixture followed by 10 s vortex. Each sample was centrifuged (14,000 × *g*) in Speedvac for 10 min without temperature to observe double meniscus layer (chloroform at the bottom). Meanwhile, each labeled empty vial was filled with anhydrate sodium sulfate to remove humidity and 150 µL insert was placed to each vial. Lastly, 130 µL of the chloroform (bottom) phase of each sample mixture was transferred to the insert in the vial, containing anhydrate sodium sulfate. The reaction scheme for C15:0 and C17:0 derivatization can be found in Figure S1, Supporting Information.

### 2.4 GC‐MS Instrumentation and Analysis Parameters

GC‐MS measurements of C15:0 and C17:0 derivatives were performed according to Zhao et al.^[^ ^31^ ^]^ In brief, GC‐MS system consisted of an Agilent 7890 B (Agilent Technologies, Palo Alto, CA, USA), equipped with an automatic liquid sampler model 7693 (Agilent Technologies, Palo Alto, CA, USA), coupled to an Agilent 7000 C single quadruple mass selective detector (Agilent Technologies, Palo Alto, CA, USA). Acquisition was carried out using MassHunter workstation software for Q (Agilent Technologies, Palo Alto, CA). The GC was fitted with a DB‐5 MS capillary column (30 m, 0.25 mm i.d., 0.25 µm film thickness; (5%‐phenyl)‐methylpolysiloxane bonded and cross‐linked; Agilent J&W Scientific, Folsom, CA) and helium was used as carrier gas at 1 mL min^−1^. One microliter of each derivatized samples was injected in split less mode injector. A glass liner ultra‐inert, split‐less, single taper, and glass wool (5190‐2293, Agilent Technologies, Palo Alto, CA, USA) was used to avoid a possible contamination with non‐volatile elements into the column. The temperature gradient was 45 °C (held for 1 min) to 260 °C at 20 °C min^−1^ and 320 °C at 40 °C min^−1^ (held for 2 min). The injection, interface, and source temperature of the mass spectrometer were 270, 270, and 220 °C, respectively. The total time for the analysis was 15.25 min. The electron impact (EI) ionization (70 eV) in the m/z range of 38–650 was used, and acquisition rate was 20 spectra s^−1^. Prepared serum samples were analyzed in a random order containing one quality control (QC) every 10 samples. QC samples were prepared by pooling 50 µL from each serum sample.

### 2.5 Data Pre‐Processing

Raw data from GC‐MS analysis were exported to MassHunter Qualitative Analysis Software (vB.07.01). The targeted metabolites were identified by comparing mass spectra and retention index in the in‐house reference library of ethyl chloroformate (ECF) derivatives. Thereafter, the dataset was exported to MassHunter Quantitative Analysis Software (vB.07.0) to perform baseline correction, smoothing, noise reduction, deconvolution, library searching, and data integration in each chromatogram from samples and the calibration standards. The responses of the sample and standards were normalized by calculating the ratio of the peak area and the corresponding area of the internal standard as the corrected area. The squared correlation coefficient (*R* ^2^) was used as a measure of linearity of the calibration curve in which the known amounts of C15:0 and C17:0 was linearly regressed against corrected area.

### 2.6 Statistical Analysis

#### 2.6.1 The Dietary Fiber Supplementation Intervention Trial (Study 1)

A sample size of 19 volunteers would be needed based the primary outcome measure of the previous dietary fiber supplementation study, detailed in supplemental methods.^[^ ^28^ ^]^ Data were checked for normality by using Shapiro‐Wilk Test. Non‐parametric data were log transformed before performing parametric analysis. Repeated measure ANOVA was performed to determine differences in serum OCFAs levels, significant effects were followed up with Fisher's least significant difference (LSD) post hoc test.

#### 2.6.2 The Highly Controlled in‐Patient Feeding Study (Study 2)

A sample size of 19 was estimated based the primary outcome measure of previous controlled inpatient feeding study, detailed in supplemental methods.^[^ ^30^ ^]^ Data were checked for normality by using Shapiro‐Wilk Test. A paired *t* test was performed to determine differences in serum OCFAs levels if data were normally distributed. Wilcoxon rank sum test was used for non‐parametric data. Partial Spearman correlations analyses were used to assess the association between dietary fiber and OCFAs, adjusted for age, gender, BMI, and dairy fat of each diet using all data. The significance was considered *p* < 0.05. Data are presented as mean ± SEM. Statistical analysis was performed using IBM SPSS Statistics v24. Graphs are presented using GraphPad Prism v5.0‐7.0.

## 3 Results

### 3.1 Targeting C15:0 and C17:0 in Serum Samples

The targeted analysis showed detectable amount of C15:0 and C17:0 in study 1 and 2. The retention time for C15:0 is 10.70 with (m/z 87.1, 43.1, 55.1, 143.1). The retention time for C17:0 is 11.73 with (m/z 87.1, 143.1, 43.1, 55.1). The calibration curves with satisfactory linearity (C15:0, *R* ^2^ = 0.996; C17:0, *R* ^2^ = 0.995) were used to quantify C15:0 and C17:0 concentrations in the study samples.

### 3.2 Validating OCFAs in Dietary Fiber Supplementation Study (Study 1)

GC‐MS targeted analysis of the OCFAs, C15:0 and C17:0 was performed on serum samples collected from 17 of the 21 volunteers that completed the study. Four participants were removed from analysis due to missing samples at any of the three‐time points. There was no evidence carry‐over effect in the main outcome measures across two supplementation period (Table S2, Supporting Information).

**Figure 3:**
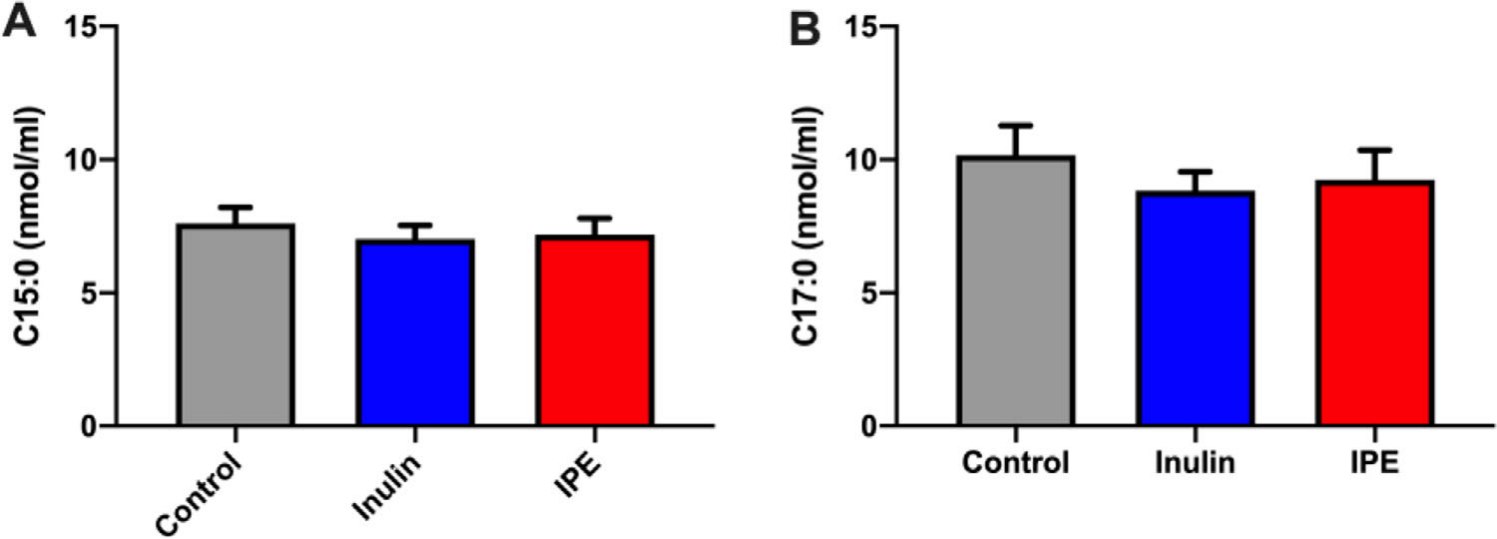
Total serum concentrations (nmol mL^−1^) of A) C15:0 and B) C17:0. The levels of circulating OCFAs were not significantly different among control, inulin, and IPE supplementations (C15:0, *p* = 0.404; C17:0, *p* = 0.288). Data are presented as mean ± SEM (*n* = 17), significance considered *p* < 0.05. SEM, standard error of the mean.

**Figure 4:**
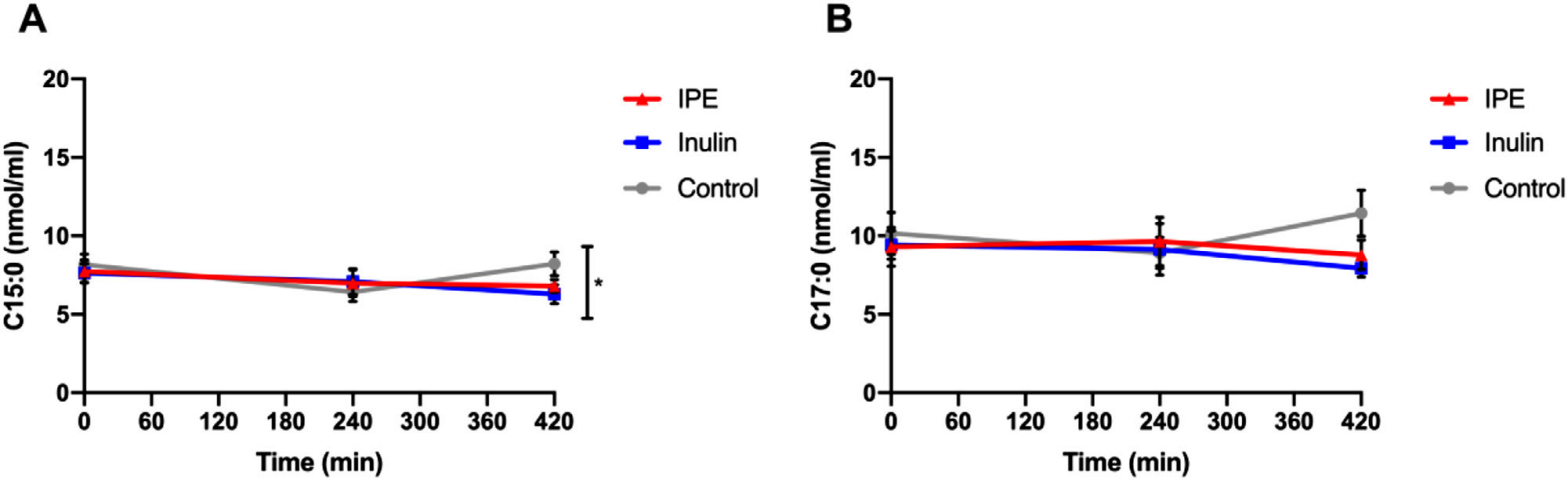
The time course profiles of postprandial serum concentrations (nmol mL^−1^) of OCFAs A) C15:0, B) C17:0 following control, inulin, and IPE supplementations. * indicate a significant decrease (*p* = 0.036) in A) C15:0 at 420 min following inulin supplementation relative to control. Data presented as mean ± SEM (*n* = 17), significance considered *p*<0.05. SEM, standard error of the mean.

**Figure 5:**
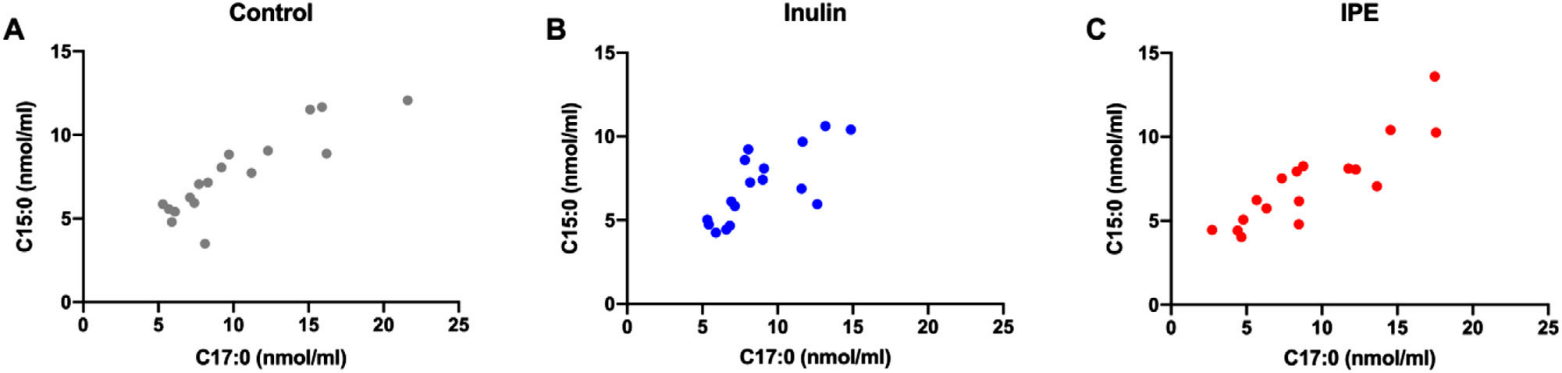
Correlations between C15:0 and C17:0 in A) control, B) inulin, and C) IPE supplementation.

Overall, there were no effects of fiber supplementations on the serum concentrations of C15:0 and C17:0, as the changes in OCFAs concentrations were not significantly different among control, inulin and IPE supplementations (C15:0, *p* = 0.404; C17:0, *p* = 0.288), (**Figure** **3**A, B). There were no differences in the levels of OCFAs over time, as the levels of OCFAs were not different between 0, 240, 420 min (C15:0, *p* = 0.074; C17:0, *p* = 0.503). The time course profiles of C15:0 and C17:0 are illustrated in **Figure** **4**A, B. A significant treatment by time interaction was observed for C15:0 (*p* = 0.036), in which serum concentrations of C15:0 was significantly decreased at time 420 min following inulin supplementation compared with control (*p* = 0.011), (Figure 4A.; Table S3–4, Supporting Information). In addition, levels of C17:0 were positively correlated with C15:0 among control, inulin and IPE supplementations (**Figure** **5**), which possibly suggests that C15:0 and C17:0 share a similar dietary origin.

### 3.3 Validating OCFAs in Controlled Inpatient Feeding Study (Study 2)

**Figure 6:**
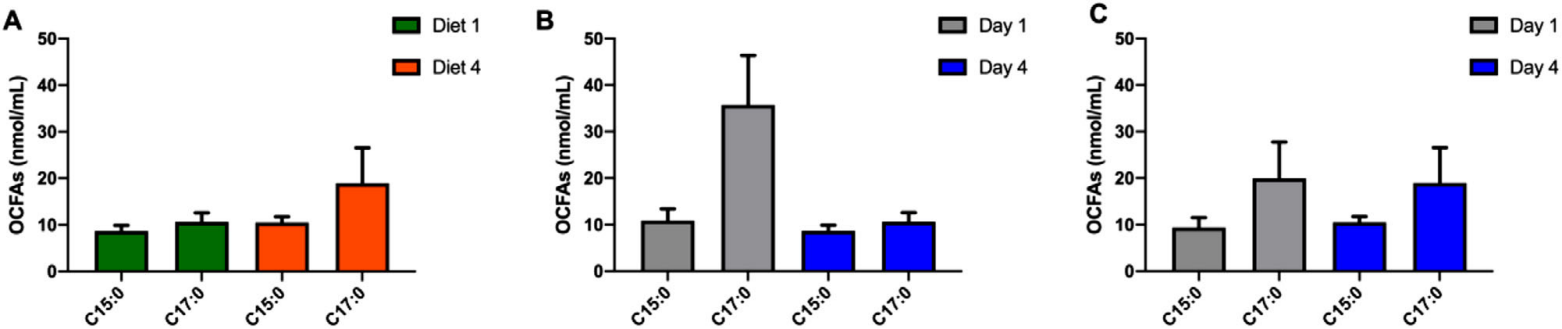
The effects of high and low fiber diet on OCFAs responses in controlled inpatient feeding study.A) Fasting serum OCFAs concentrations measured after 4‐day adherence to high fiber (Diet 1, higlighted in green) and low fiber diet (Diet 4, highlighted in orange), there were no significant differences (C15:0, *p* = 0.091; C17:0, *p* = 0.199). B) Fasting serum OCFAs levels measured before (at Day 1, highlighted in grey) and after (at Day 4, highlighted in blue) following high fiber diet, there were no significant differences (C15:0, *p* = 0.445; C17:0, *p* = 0.326). C) Fasting serum OCFAs levels measured before (Day 1, highlighted in grey) and after (Day 4, highlighted in blue) following low fiber diet, there were no significant differences (C15:0, *p* = 0.446; C17:0, *p* = 0.326). Data presented as mean ± SEM (*n* = 19), significance considered *p*<0.05. SEM, standard error of the mean.

No difference on OCFAs concentrations measured in fasting serum samples were found at baseline while comparing two intervention groups (C15:0, *p* = 0.586; C17:0, *p* = 0.196). Fasting serum OCFAs concentrations were not significantly different (C15:0, *p* = 0.091; C17:0, *p* = 0.199) after 4 days of adherence to high fiber or low fiber diet despite the substantial difference in fiber intake (Diet 1 = 45.1 g per day, Diet 4 = 13.6 g per day), (**Figure** **6**A; Table S5–6, Supporting Information).

Additionally, there were no significant changes in OCFAs concentrations before and after adopting either low fiber (C15:0, *p* = 0.446; C17:0, *p* = 0.326) or high fiber diet (C15:0, *p* = 0.445; C17:0, *p* = 0.326) (Figure 6B, C; Table S5–6, Supporting Information).

Furthermore, OCFAs levels were not significantly different between high and low fiber diets measured after breakfast and after lunch (C15:0, *p* = 0.286, *p* = 0.586; C17:0, *p* = 0.744, *p* = 0.433 respectively). However, the level of C15:0 was significantly increased (*p* = 0.036) after dinner following low fiber diet (Table S5–6, Supporting Information).

Further analyses were performed to investigate the potential association between dietary fiber and OCFAs. Correlation analyses adjusted for age, gender, BMI, and dairy fat have shown that C15:0 and C17:0 fasting serum levels were not correlated with dietary fiber (C15:0, *r* = −0.08, *p* = 0.50; C17:0, *r* = 0.13, *p* = 0.27).

## 4 Discussion

In the present work, we used a targeted approach to validate OCFAs, specifically, C15:0 and C17:0 as biomarkers of dietary fiber intake in two independent dietary studies following the validation scheme proposed by Dragsted et al.^[^ ^20^ ^]^ OCFAs were first assessed in a dietary fiber supplementation study (study 1), which shared a similar study design to the previous investigation.^[^ ^23^ ^]^ In particular, a short‐term accumulated dietary effect was built by repeated 6‐day fiber supplementation leading to an acute feeding investigation on day‐7. In study 1, dietary fiber supplementations were incorporated into habitual diet to capture the dietary variations as seen in free‐living conditions in order to assess the plausibility and robustness. Given that dietary compliance monitoring is inherently challenging as result of misreporting, OCFAs were further assessed under a highly controlled 4‐day inpatient feeding regime to investigate biomarker reliability (study 2). We used a previously validated and published analytical method that exhibited good reproducibility and stability for quantitative microbiome metabolomics.^[^ ^31^ ^]^ The analysis of the same method has been performed in two laboratories in two different studies.

Collectively in both studies, increased in dietary fiber consumption did not lead to increased circulating OCFAs concentrations. In study 1, it is possible that the amount of propionate (2.36 g propionate in 10 g per day IPE) or inulin (10 g per day) posed negligible effect on OCFAs concentrations. In contrast, a much higher dose of either sodium propionate (6 g per day) or inulin (30 g per day) were provided as supplements in previous work.^[^ ^23^ ^]^ Therefore, OCFAs might not be sufficiently sensitive as fiber biomarkers for lower dose of supplementation. However, the selected dosages in the current study were thought to be physiologically feasible. It is estimated that 10 g IPE (2.36 g propionate) would result in 2.5‐fold increase in daily propionate production.^[^ ^29^ ^]^ Whereas 10 g of inulin in addition to participants daily fiber intake should achieve the recommended intake of 30 g per day.^[^ ^4^ ^]^ According to the national diet and nutritional survey, the average dietary fiber intake for adults in the UK was 19.7 g, and less than 20 g globally.^[^ ^32^ ^]^ In study 2, no significant differences in fasting OCFAs concentrations were found between a high fiber (45.1 g per day) and a low fiber diet (13.6 g per day) after 4‐day inpatient period. OCFAs could be related to inulin or oral propionate intake, however, our work suggests that non‐physiological amounts would be needed for these changes. Here, the lack of elevation in OCFAs concentrations in either of the two studies calls into question the assumption of the underlying relationship between dietary fiber and OCFAs.

Indeed, the most fundamental criterion for biomarker validation is the presence of substantial evidence to support the relationship between dietary fiber and OCFAs, i.e., the plausibility. Prior investigation has proposed that high intake of dietary fiber leads to elevated microbial fermentation and increased propionate production.^[^ ^23^ ^]^ Propionate is further converted to propionyl‐CoA which competes with acetyl‐CoA for OCFAs biosynthesis.^[^ ^23^ ^]^ In accordance, investigations into inter‐organ SCFAs metabolism has emphasized that systemic propionate concentration may not be a true representation of colonic propionate production because propionate undergoes substantial uptake and metabolism by the liver.^[^ ^28^, ^33^, ^34^, ^35^ ^]^ Our previous work on the current dietary fiber supplementation study (study 1) have demonstrated that despite dietary fiber supplementations circulating propionate levels were not significantly different among interventions.^[^ ^28^ ^]^ Therefore, gut derived propionate could be used for hepatic OCFAs biosynthesis as previously proposed.^[^ ^23^ ^]^ To unravel the relationship between dietary fiber, gut microbial fermentation and OCFAs production, we have focused on OCFAs measured at baseline, 240 and 420 min (study 1). The reason for selecting these time points is because the previous investigation in study 1 have shown that breath H_2_ concentrations, a marker of gut bacterial fermentation, were significantly elevated above baseline concentrations following intake of inulin and IPE between 240 and 420 min.^[^ ^28^ ^]^ Moreover, following intake of a ^13^C labeled IPE, a significant increase in propionate ^13^C enrichment is observed in the peripheral circulation between 240 and 420 min, revealing that the bound propionate from IPE is absorbed from the gut and is available systemically in this period.^[^ ^29^ ^]^ In study 1, despite the ensured gut microbial fermentation and the delivery of propionate into the colon, no positive changes in OCFAs concentrations were observed as result of inulin or IPE supplementations (Figure 3). Interestingly, a significant decrease in C15:0 at 420 min was observed following inulin supplementation relative to control (Figure 4A). Previous data have shown that SCFAs produced from inulin result in a 74:16:10 ratio of acetate: propionate: butyrate and that 10 g IPE produces a ratio of 25:69:6, respectively.^[^ ^29^, ^36^ ^]^ Therefore, it could be hypothesized that inulin is more acetogenic than propiogenic, which possibly leads to greater production of acetyl‐CoA as substrate for even‐chain fatty acid.

As suggested in the literature, C15:0 shows direct correlation with dietary C15:0 intake, such as from dairy fat.^[^ ^24^, ^25^, ^26^, ^27^, ^37^, ^38^ ^]^ In study 2, correlations analyses adjusted for age, gender, BMI, and dairy fat showed that fasting serum levels of OCFAs were not correlated with dietary fiber. Our observation is in agreement with a recent study where plasma C15:0 level did not show correlation with dietary fiber.^[^ ^39^ ^]^ In addition,dairy fat, fatty fish, dietary fiber, olive oil, and protein have been indicated as dietary precursors for C17:0.^[^ ^40^, ^41^ ^]^ The utility of C17:0 as dietary biomarker is limited due to this non‐specificity. Evidence from in vitro investigation has suggested that the overall circulating level of C17:0 is unmatched with the amount produced via propionyl‐CoA elongation.^[^ ^38^ ^]^ In the mouse model, the OCFAs levels were not influenced by the presence or absence of gut microbiota.^[^ ^38^ ^]^ Indeed, the gut microbiota plays an important role in establishing the relationship between dietary fiber and OCFAs.

With respect to the two studies defined here, the relative short duration could be considered as a limitation. The complex relationship between dietary fiber and OCFAs possibly suggest a longer follow up is needed to fully capture the pattern of response, especially when postprandial kinetics and optimal measurement time is uncertain. In addition, our work measured OCFAs in absolute concentrations, which indeed was not exactly comparable to the previous study. Lastly, the clinical trials were not specifically designed to assess the effect of dietary fiber on OCFAs. However, both studies serve as sound models for biomarker validation.

## 5 Conclusion

Suitability of OCFAs as candidate biomarkers in free living condition was investigated by incorporating fiber supplementations into the habitual diet in study 1, further assessment of the OCFAs reliability were assessed in a highly controlled setting in study 2. Dietary fiber supplementations or having consumed a high fiber diet did not increase circulating OCFAs. Temporal relation of OCFAs and dietary fiber was not observed in post meal monitoring after repeated supplementations despite the fact that microbial fermentation and the delivery of propionate was assured. The time response was also not evident within 4‐day inpatient stays. Our work has generated new insight on the applicability of OCFAs as fiber biomarkers and highlighted the importance of dietary biomarker validation studies to critically evaluate candidate biomarkers prior application.

## Conflict of Interest

G.F. is lead for the Imperial Nestlé Collaboration and reports personal fees from Unilever, both outside the submitted work.

## Author Contributions

I.G.‐P., E.S.C., Y.W.W. conceptualized the study and wrote the manuscript. Y.W.W., I.G.‐P performed sample analysis. Y.W.W., J.M.P., I.G.‐P., E.S.C. contributed to data analysis. I.G.‐P., E.S.C., and G.F. ran the clinical trials. All authors read and approved the final manuscript, and approved the final submitted version. G.F., I.G.‐P. assumes responsibility for the completeness and accuracy of the data and analyses, and for adherence to the study protocol.

## Supporting information

Supporting information

## Data Availability

The data that support the findings of this study are available from the corresponding author upon reasonable request.
