# Supplementary material for: Odd Chain Fatty Acids Are Not Robust Biomarkers for Dietary Intake of Fiber
Source: Mol Nutr Food Res. 2021 Oct 22;65(22):2100316. doi: 10.1002/mnfr.202100316 (PMC11475553; doi:10.1002/mnfr.202100316)
Supplement: Supplementary file 1 — Supporting information [file MNFR-65-2100316-s001.docx]

**Supporting information**

**Supplemental Methods**

*Recruitment and eligibility for study 1*

Participants were recruited from a database of healthy volunteers at the UK National Institute for Health Research (NIHR)/Wellcome Trust Imperial Clinical Research Facility (CRF) and poster advertisements. A sample size of 19 volunteers would be needed based on the power calculator estimation. The sample size was determined by the primary outcome measure of previous supplementation feeding study. In which, a power of 80%, α =0.05, to detect a difference in mean plasma PYY concentration of 16 pmol/L with a standard deviation of 23 in each group. 23 healthy participants were enrolled into the study after screening, with exclusion criteria set as weight change of ≥ 3 kg in the preceding 2 months, current smokers, excess alcohol intake, substance abuse, any chronic illness or GI disorder, pregnancy and use of medications likely to interfere with energy metabolism, appetite regulation and hormonal balance, including: anti-inflammatory drugs or steroids, antibiotics, androgens, phenytoin, erythromycin or thyroid hormones. Two participants dropout from the study before attending the first study visit, 21 participants completed the clinical trial (Byrne et al., 2019).

*Recruitment and eligibility for study 2*

352 participants were recruited from a database of healthy volunteers at the UK National Institute for Health Research (NIHR)/Wellcome Trust Imperial Clinical Research Facility (CRF). After first screening, 300 volunteers left (21–65 years, BMI of 20–35 kg/m^2^) were contacted with letters of invitations. 78 volunteers who accepted the invitations were screened initially by email or telephone, and subsequently on site. 20 healthy participants were finally enrolled into the study after screening, with exclusion criteria set as participates who had clinically significant illness, reported weight loss or gain of equal to or more than 3kg in the last 2 months, and participates who were smokers, substance abusers, or presented any abnormalities on physical examination, electrocardiography or screening blood test. Women volunteers were excluded if they were pregnant or breastfeeding. With one dropout due to schedule conflict, 19 participants completed the clinical trial. As this study was first in human trial of metabolic profiling in controlled inpatient feeding setting, no formal power calculation could be taken. To provide a basis for sample size calculation, data on urinary proline betaine were used as a representative marker for nutritional intake (Heinzmann et al., 2010), in which urinary concentration of this metabolite would rise by 50 μmol/L with each incremental rise in fruit intake (ie, pieces of fruit) in the experimental setting. Therefore, with an SD of 40 μmol/L, assuming a power of 0.95 and an alpha of 0.05 to detect a difference of 50 μmol/L, 12 participants would be needed. 30 people were targeted for recruiting, with the aim of having a cohort of roughly 20 people. All 19 participants who completed the study were included in the analysis (Garcia-Perez et al., 2017).

**Participant Flow Chart**

Assessed for eligibility (n=27)

**Study 1 The dietary fiber supplementation intervention trial**

Excluded (n=4)

Not meeting inclusion criteria (n=3)

Declined to participate (n=1)

Randomized (n=23)

Allocated to intervention on 1^st^ supplementation visit (n=13)

Allocated to intervention on 2^nd^ supplementation visit (n=10)

Drop-outs (n=2)

Allocated to intervention on 1^st^ supplementation visit (n=1)

Allocated to intervention on 2^nd^ supplementation visit (n=1)

Analysed (n=21)

**Participant Flow Chart**

**Study 2 The highly controlled in-patient feeding study**

352 healthy volunteers in database

Excluded (n=52)

Aged >65 years (n=20)

Aged <20 years (n=10)

BMI >35 kg/m^2^ (n=16)

BMI <20 kg/ m^2^ (n=6)

Sent letters of invitation (n=300)

No response (n=222)

Screened by email or telephone (n=78)

Declined participation (n=42)

Not eligible (n=10)

On prescription medication (n=8)

BMI >35kg/m^2^ (n=2)

Screened on site (n=26)

Declined participation (n=5)

Not eligible (n=1)

BMI >35kg/m^2^ (n=1)

Randomized (n=20)

Withdrawn because of scheduling conflicts (n=1)

Completed study and assessed (n=19)

**Supplemental Table 1**. Dietary information and nutritional profile of 4 types of diets in highly controlled in-patient feeding study. Diet 1: High fiber (45.1g/day) and Diet 4: Low fiber (13.6 g/day).

| *Diet 1* | | *Diet 2* | | *Diet 3* | | | *Diet 4* | |
| --- | --- | --- | --- | --- | --- | --- | --- | --- |
| *Food* | *Serving*  *(g)* | *Food* | *Serving*  *(g)* | | *Food* | *Serving*  *(g)* | *Food* | *Serving (g)* |
| ***Breakfast (9:00)*** |  |  |  | |  |  |  |  |
| *Whole wheat cereal* | *60* | *Sugar coated cereal* | *15* | | *Sugar coated cereal* | *30* | *Sugar coated cereal* | *60* |
| *Semi-skimmed milk* | *150* | *Whole milk* | *50* | | *Whole milk* | *100* | *Whole milk* | *150* |
| *Wholemeal bread, toasted* | *60* | *White bread, toasted* | *20* | | *White bread, toasted* | *40* | *White bread, toasted* | *60* |
| *Margarine, polyunsaturated* | *10* | *Butter* | *2.5* | | *Butter* | *7.5* | *Butter* | *10* |
| *Egg, hard boiled* | *60* | *Whole wheat cereal* | *40* | | *Whole wheat cereal* | *20* |  |  |
|  |  | *Semi-skimmed milk* | *100* | | *Semi-skimmed milk* | *50* |  |  |
|  |  | *Wholemeal bread, toasted* | *40* | | *Wholemeal bread, toasted* | *20* |  |  |
|  |  | *Margarine, polyunsaturated* | *7.5* | | *Margarine, polyunsaturated* | *2.5* |  |  |
|  |  | *Egg, hard boiled* | *30* | |  |  |  |  |
| ***Morning Snack (11:00)*** |  |  |  | |  |  |  |  |
| *Apple, Granny Smith* | *150* | *Apple, Granny Smith* | *100* | | *Low fat yoghurt* | *125* | *Greek yoghurt* | *125* |
|  |  |  |  | | *Apple, Granny Smith* | *50* |  |  |
| ***Lunch (13:00)*** |  |  |  | |  |  |  |  |
| *Salmon, steamed* | *150* | *Cod, steamed* | *150* | | *Sausage casserole* | *125* | *Pork sausages, fried* | *125* |
| *Jacket potato* | *200* | *New potato* | *200* | | *Oven chips, baked* | *150* | *Potato waffles, grilled* | *120* |
| *Garden peas, boiled* | *60* | *Garden peas, boiled* | *30* | | *Garden peas, boiled* | *15* | *Cola* | *330* |
| *Carrots, boiled* | *60* | *Carrots, boiled* | *30* | | *Carrots, boiled* | *15* |  |  |
| *Broccoli, boiled* | *100* | *Broccoli, boiled* | *75* | | *Broccoli, boiled* | *50* |  |  |
|  |  | *Diet cola* | *330* | | *Cola* | *330* |  |  |
| ***Afternoon Snack (15:00)*** |  |  |  | |  |  |  |  |
| *Grapes* | *150* | *Dark Chocolate* | *50* | | *Milk Chocolate* | *22.5* | *Milk Chocolate* | *45* |
|  |  | *Grapes* | *100* | | *Dark Chocolate* | *25* |  |  |
|  |  |  |  | | *Grapes* | *50* |  |  |
| ***Dinner (18:00)*** |  |  |  | |  |  |  |  |
| *Chicken breast, grilled* | *125* | *Chicken breast, fried* | *125* | | *Beef burgers, grilled* | *100* | *Beef burgers, fried* | *100* |
| *Whole wheat pasta* | *150* | *White pasta* | *150* | | *Oven chips, baked* | *150* | *Potato waffles, grilled* | *120* |
| *Peppers* | *80* | *Peppers* | *40* | | *Baked beans in tomato sauce* | *150* | *Processed cheese* | *30* |
| *Onion* | *40* | *Onion* | *20* | | *Cheddar cheese* | *40* | *Tomatoes* | *100* |
| *Tomato pasta sauce* | *150* | *Tomato pasta sauce* | *150* | | *Diet Cola* | *330* | *Cola* | *330* |
|  |  | *Diet cola* | *330* | |  |  |  |  |
| ***Evening Snack (21:00)*** |  |  |  | |  |  |  |  |
| *Wholemeal bread, toasted* | *80* | *White bread, toasted* | *40* | | *White bread, toasted* | *40* | *White bread, toasted* | *80* |
| *margarine, polyunsaturated* | *10* | *Butter* | *2.5* | | *Butter* | *7.5* | *Butter* | *10* |
|  |  | *Wholemeal bread, toasted* | *40* | | *Wholemeal bread, toasted* | *40* |  |  |
|  |  | *Margarine, polyunsaturated* | *7.5* | | *Margarine, polyunsaturated* | *2.5* |  |  |
| ***Macronutrient contents and dietary features*** | |  |  | |  |  |  |  |
| *Energy (kcal)* | *2260* | *2259* | | | *2427* | | *2490* | |
| *Energy density (kcal/g)* | *1.2* | *1·5* | | | *1·6* | | *1·9* | |
| *Protein (%)* | *24%* | *22%* | | | *16%* | | *13%* | |
| *CHO (%)* | *51%* | *51%* | | | *46%* | | *44%* | |
| *Fat (%)* | *23%* | *24%* | | | *35%* | | *42%* | |
| *Total sugar (g)* | *14* | *18* | | | *22* | | *25* | |
| *Saturated fatty acids (g)* | *5* | *7* | | | *19* | | *20* | |
| *Monounsaturated fatty acids (g)* | *8* | *6* | | | *14* | | *12* | |
| *Polyunsaturated fatty acids (g)* | *8* | *5* | | | *4* | | *2* | |
| *Total trans fatty acids (g)* | *0.5* | *0·5* | | | *1* | | *1* | |
| *Fiber (g)* | *45.9* | *32·1* | | | *31·5* | | *13·6* | |
| *Sodium (mg)* | *2367* | *2261* | | | *3812* | | *3066* | |
| *Fruit and vegetables (g)* | *600* | *300* | | | *180* | | *100* | |
| *DASH score* | *37* | *30* | | | *24* | | *11* | |

**Supplemental Table 2.** The effect of supplementation order on primary outcome measures

|  | Supplementation Period 1 | Supplementation Period 2 | P Value |
| --- | --- | --- | --- |
| C15 | 6.626 ± 0.488 | 6.901 ± 0.589 | 0.256 |
| C17 | 8.394 ± 0.670 | 8.782 ± 1.053 | 0.571 |

Data presented as mean ± SEM (n=17), significance considered p<0.05.

**Supplemental Table 3**. The OCFAs responses in acute dietary fiber supplementation investigation

| Treatment | Units | Control | Inulin | IPE | Repeated measure ANOVA (P values) |  |  |
| --- | --- | --- | --- | --- | --- | --- | --- |
|  |  |  |  |  | Treatment | Time | Time x Treatment |
| C15 | nmol/ml | 7.185 ± 0.571 | 6.626 ± 0.488 | 6.901 ± 0.589 | p=0.404 | p=0.074 | p=0.036* |
| C17 | nmol/ml | 9.665 ± 1.064 | 8.394 ± 0.670 | 8.782 ± 1.053 | p=0.288 | p=0.503 | p=0.430 |

Data presented as mean ± SEM (n=17), significance considered p<0.05, * indicates a significant difference

**Supplemental Table 4**. C15 post hoc analysis for the evaluation of pairwise differences in treatment by time in acute supplementation intervention trial

| Treatments |  |  | P values | Treatments |  |  | P values |
| --- | --- | --- | --- | --- | --- | --- | --- |
| Control, baseline | vs. | Inulin, baseline | 0.405 | Control, 240 min | vs. | Inulin, 240 min | 0.240 |
| Control, baseline | vs. | IPE, baseline | 0.526 | Control, 240 min | vs. | IPE, 240 min | 0.318 |
| Inulin, baseline | vs. | IPE, baseline | 0.898 | Inulin, 240 min | vs. | IPE, 240 min | 0.889 |
| Control, 420 min | vs. | Inulin, 420 min | 0.011* | Inulin, 420 min | vs. | IPE, 420 min | 0.100 |
| Control, 420 min | vs. | IPE, 420 min | 0.157 |  |  |  |  |

significance considered p<0.05, * indicates a significant difference

**Supplemental Table 5.** The OCFAs concentrations following diets with differing fiber contents in highly controlled in-patient feeding study

| Diets | OCFAs | Units | Day 1 | Day 4 |  | Day 3 |  |  |
| --- | --- | --- | --- | --- | --- | --- | --- | --- |
|  |  |  | Fasting | Fasting |  | After Breakfast | After Lunch | After Dinner |
| Diet 1 (high) | C15 | nmol/l | 10.916± 2.470 | 8.669 ± 1.243 |  | 4.985 ± 0.275 | 4.969± 0.296 | 5.559 ± 0.326 |
| Diet 1 | C17 | nmol/l | 35.734± 10.640 | 10.640 ± 1.958 |  | 9.373 ± 1.434 | 7.715 ± 1.519 | 8.100 ± 1.063 |
|  |  |  |  |  |  |  |  |  |
| Diet 4(low) | C15 | nmol/l | 9.385 ± 2.119 | 10.522 ± 1.249 |  | 5.384 ± 0.277 | 5.163± 0.203 | 6.281 ± 0.383 |
| Diet 4 | C17 | nmol/l | 19.923 ± 7.821 | 18.995 ± 7.507 |  | 8.832± 1.153 | 6.461± 1.354 | 10.976 ± 1.709 |

Data presented as mean ± SEM (n=19). Diet 1, high fiber diet; Diet 4, low fiber diet.

**Supplemental Table 6**. Statistical comparisons of fasting and postprandial OCFAs levels in highly controlled in-patient feeding study

| Fasting Day 1 and Day 4 | C15 | C17 |
| --- | --- | --- |
| Day 1 Diet 1 vs. Day 1 Diet 4 | P=0.586 | p=0.196 |
| Day 4 Diet 1 vs. Day 4 Diet 4 | p=0.091 | p=0.199 |
|  |  |  |
| Diet 1 Day 1 vs. Diet 1 Day 4 | P=0.445 | P=0.326 |
| Diet 4 Day 1 vs. Diet 4 Day 4 | P=0.446 | P=0.326 |
|  |  |  |
| Day 3 | C15 | C17 |
| After breakfast, Diet 1 vs. Diet 4 | P=0.286 | P=0.744 |
|  |  |  |
| After Lunch, Diet 1 vs. Diet 4 | P=0.586 | P= 0.433 |
|  |  |  |
| After dinner, Diet 1 vs. Diet 4 | p =0.036* | p = 0.286 |

Significance considered p<0.05, Diet 1, high fiber diet; Diet 4, low fiber diet. * indicates a significant

Difference


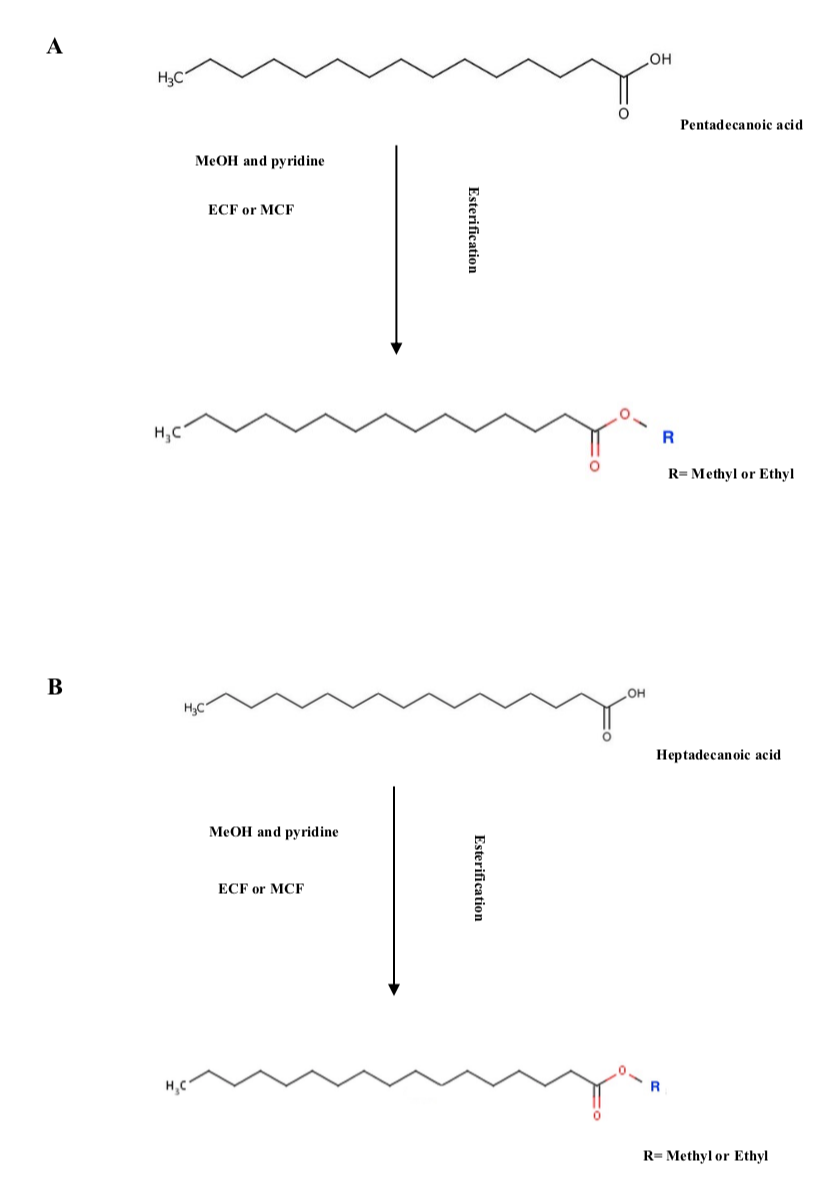


**Supplemental Figure 1.** The reaction scheme for A) pentadecanoic acid and B) heptadecanoic acid treated with Ethyl chloroformate (ECF) or Methyl chloroformate (MCF) derivatization. Simultaneous esterification of carboxylic group yields alkyl ester that are more amenable in the GC.

STROBE Statement—checklist of items that should be included in reports of observational studies

|  | **Item No** | **Recommendation** | **Page  No** |
| --- | --- | --- | --- |
| **Title and abstract** | 1 | (*a*) Indicate the study’s design with a commonly used term in the title or the abstract | 1-2 |
|  |  | (*b*) Provide in the abstract an informative and balanced summary of what was done and what was found | 2 |
| **Introduction** | | | |
| Background/rationale | 2 | Explain the scientific background and rationale for the investigation being reported | 3-4 |
| Objectives | 3 | State specific objectives, including any prespecified hypotheses | 3-4 |
| **Methods** | | | |
| Study design | 4 | Present key elements of study design early in the paper | 4-9 |
| Setting | 5 | Describe the setting, locations, and relevant dates, including periods of recruitment, exposure, follow-up, and data collection | Supplementary  Data 1-3 |
| Participants | 6 | (*a*) *Cohort study*—Give the eligibility criteria, and the sources and methods of selection of participants. Describe methods of follow-up  *Case-control study*—Give the eligibility criteria, and the sources and methods of case ascertainment and control selection. Give the rationale for the choice of cases and controls  *Cross-sectional study*—Give the eligibility criteria, and the sources and methods of selection of participants | Supplementary  Data 1-3 |
|  |  | (*b*) *Cohort study*—For matched studies, give matching criteria and number of exposed and unexposed  *Case-control study*—For matched studies, give matching criteria and the number of controls per case | Not Applicable |
| Variables | 7 | Clearly define all outcomes, exposures, predictors, potential confounders, and effect modifiers. Give diagnostic criteria, if applicable | 4-9 |
| Data sources/ measurement | 8* | For each variable of interest, give sources of data and details of methods of assessment (measurement). Describe comparability of assessment methods if there is more than one group | 4-9 |
| Bias | 9 | Describe any efforts to address potential sources of bias | Not Applicable |
| Study size | 10 | Explain how the study size was arrived at | Supplementary  Data 1-3 |
| Quantitative variables | 11 | Explain how quantitative variables were handled in the analyses. If applicable, describe which groupings were chosen and why | Not Applicable |
| Statistical methods | 12 | (*a*) Describe all statistical methods, including those used to control for confounding | 8 |
|  |  | (*b*) Describe any methods used to examine subgroups and interactions  (*c*) Explain how missing data were addressed  (*d*) *Cohort study*—If applicable, explain how loss to follow-up was addressed  *Case-control study*—If applicable, explain how matching of cases and controls was addressed  *Cross-sectional study*—If applicable, describe analytical methods taking account of sampling strategy  (*e*) Describe any sensitivity analyses | Not Applicable |

Continued on next page

| **Results** | | | |
| --- | --- | --- | --- |
| Participants | 13* | (a) Report numbers of individuals at each stage of study—eg numbers potentially eligible, examined for eligibility, confirmed eligible, included in the study, completing follow-up, and analysed  (b) Give reasons for non-participation at each stage  (c) Consider use of a flow diagram | Supplementary  Data 1-3 |
| Descriptive data | 14* | (a) Give characteristics of study participants (eg demographic, clinical, social) and information on exposures and potential confounders | Supplementary  Data 1-3 |
|  |  | (b) Indicate number of participants with missing data for each variable of interest | 9 |
|  |  | (c) *Cohort study*—Summarise follow-up time (eg, average and total amount) | Not Applicable |
| Outcome data | 15* | *Cohort study*—Report numbers of outcome events or summary measures over time  *Case-control study—*Report numbers in each exposure category, or summary measures of exposure  *Cross-sectional study—*Report numbers of outcome events or summary measures | 9-10 |
| Main results | 16 | (*a*) Give unadjusted estimates and, if applicable, confounder-adjusted estimates and their precision (eg, 95% confidence interval). Make clear which confounders were adjusted for and why they were included | 9 |
|  |  | (*b*) Report category boundaries when continuous variables were categorized | Not Applicable |
|  |  | (*c*) If relevant, consider translating estimates of relative risk into absolute risk for a meaningful time period | Not Applicable |
| Other analyses | 17 | Report other analyses done—eg analyses of subgroups and interactions, and sensitivity analyses | Not Applicable |
| **Discussion** | | | |
| Key results | 18 | Summarise key results with reference to study objectives | 9-11 |
| Limitations | 19 | Discuss limitations of the study, taking into account sources of potential bias or imprecision. Discuss both direction and magnitude of any potential bias | 13 |
| Interpretation | 20 | Give a cautious overall interpretation of results considering objectives, limitations, multiplicity of analyses, results from similar studies, and other relevant evidence | 11-14 |
| Generalisability | 21 | Discuss the generalisability (external validity) of the study results | Not Applicable |
| **Other information** | | | |
| Funding | 22 | Give the source of funding and the role of the funders for the present study and, if applicable, for the original study on which the present article is based | 14-15 |

*Give information separately for cases and controls in case-control studies and, if applicable, for exposed and unexposed groups in cohort and cross-sectional studies.

**Note:** An Explanation and Elaboration article discusses each checklist item and gives methodological background and published examples of transparent reporting. The STROBE checklist is best used in conjunction with this article (freely available on the Web sites of PLoS Medicine at http://www.plosmedicine.org/, Annals of Internal Medicine at http://www.annals.org/, and Epidemiology at http://www.epidem.com/). Information on the STROBE Initiative is available at www.strobe-statement.org.
